# COVID-19 and uncertainty spillovers in Indian stock market

**DOI:** 10.1016/j.mex.2020.101199

**Published:** 2020-12-23

**Authors:** Biplab Kumar Guru, Amarendra Das

**Affiliations:** a Department of Humanities and Social Sciences, Indian Institute of Technology Kharagpur, West Bengal, 721302, India; b Reader F, School of Humanities and Social Sciences, National Institute of Science Education and Research, Bhubaneswar-752050, India; Homi Bhabha National Institute, Anushakti Nagar, Mumbai, India

**Keywords:** BSE India, Volatility spillovers, Diebold-Yilmaz, COVID-19

## Abstract

In this paper, we have examined the impact of COVID-19 on the volatility spillovers among ten major sector indices listed in BSE India. We found that total volatility spillovers reached 69% during COVID-19. Energy sector followed by oil & gas were the major net volatility transmitters.

• COVID-19 has magnified the volatility spillovers in the stock market.
• Socks to energy sector significantly spills over to other sectors.
• FMCG remains the largest net recipient of the volatility spillovers from other sectors.

## Graphical abstract

**Figure:**
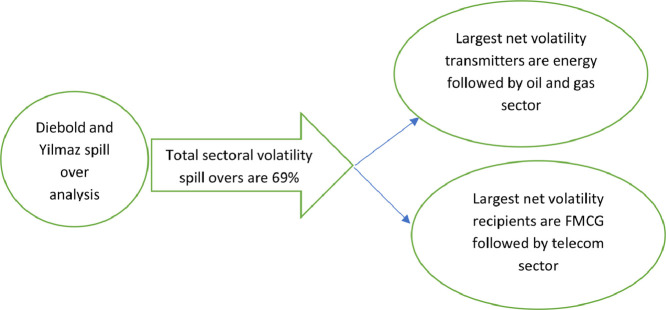

Specifications table

**Table:** 

Subject area:	Economics and Finance
More specific subject area:	*Financial Macroeconomics*
Method name:	Diebold and Yilmaz [7] Spillover Index
Name and reference of original method:	*F. X. Diebold, K. Yilmaz Measuring financial asset return and volatility spillovers, with application to global equity markets Economic Journal 119 (2009) pp. 158-171,*10.1111/j.1468-0297.2008.02208.x
Resource availability:	

## Introduction

This paper attempts to identify the sectors that were potential transmitters/recipients of the shock during COVID-19 in India. To measure volatility spillovers, we have employed Diebold and Yilmaz [7] connectedness methodology which is widely applied in the literature concerning risk-management. The connectedness approach formulated and developed by Diebold and Yilmaz [7] has been found superior to other correlation-based approaches such as pairwise correlation by Engle and Kelly [8], CoVaR approach Adrian and Brunnermeier [2] and the marginal expected shortfall approach [1].

Diebold and Yilmaz [6] show that volatility spillovers across US stock, bond, foreign exchange and commodities markets were limited until the global financial crisis (GFC). But in the aftermath of the collapse of the Lehman Brothers in September 2008, volatility spillovers were from stock market to other markets. Bala and Premaratne [4] find that shocks to the stock market of Singapore significantly transmit volatility to the stock markets of US, Hong Kong, and Japan. Similarly, Alotaibi and Mishra [3] show significant return spillovers from Saudi Arabia and US to Gulf cooperation council stock markets.

In the present context, regulatory lockdowns due to COVID-19 across the world has severely affected both real and financial sectors. The frequency of shock transmission in India has gone up substantially which have led to greater volatility co-movement. Identification and measurement of volatility spillovers helps to monitor the sectoral interdependencies, diversify the risk and smoothen the effect of shock transmission [29]. COVID-19 led to the emergence of a new set of literature on market connectedness.

Increasing COVID-19 cases created liquidity crunch in the emerging markets [11]. COVID-19 has significantly increased the economic policy uncertainty in China and Korea [14]. Another study by Iyke [15] shows that US gas and oil firms heterogeneously respond to the COVID-19 pandemic while the latter accounts for 28% of returns and 27% of return volatility. The pandemic severely affected the returns of most of the 11 listed US energy firms. A positive co-movement between returns on stock and oil price was noticed during COVID-19 which suggests a negative effect of declining oil prices on the stock market [25]. For India, there was a unidirectional causal nexus between foreign portfolio investment flows and stock returns owing to COVID-19 [24]. COVID-19 as compared to demonetization and the GST had severe adverse effect on the stock returns in India [19]. Although stock markets temporarily over-reacted to COVID-19 shock but gradually markets have consolidated [23].

COVID-19 was shown to exert a transitory effect on the Nikkei 225 index, but the effect of shocks on Kospi and Chinese CSI 300 index are permanent [9]. Also, Narayan [20] showed a transitory effect of COVID-19 on exchange rate of Japanese Yen against US dollar. The stock market volatility in Asian region comprising Hong Kong, Japan, Russia, Singapore, and South Korea has significant and positive association with stock market volatility at country-level during the COVID-19 period as against pre-COVID-19 levels [27]. And, such a relationship is more pronounced for Singapore. An interesting study by Salisu and Sikiru [26] shows that Asia-Pacific Islamic stocks can be a potential hedge during the pandemic.

Many studies have focused on COVID-19 effects for China. COVID-19 severely affected aviation, tourism, and other service sectors in China, however, sectors like new infrastructure, Chinese patent medicine, and internet industries scaled new heights [12]. Market performance of Chinese industries like transportation, mining, electricity & heating, and environment were adversely affected while sectors like manufacturing, information technology, education, and healthcare were immune to COVID-19 [13]. Private firms than state owned and foreign firms in China were disproportionately affected [10], and smaller firms were comparatively more exposed to COVID-19 [28]. Similarly, industries having high institutional investors and greater vulnerability to the virus were more affected [32].

Chen et al. [5] and Wang et al. [31] analysed the negative impact of COVID-19 on bitcoin returns and china's growth rate of insurance premium, density, and depth, respectively. Iyke [16] show that COVID-19 showed better predictive power over exchange rate volatility and returns for one and five day ahead forecast horizon, respectively. Global trade connectivity hampered due to COVID-19 but China's central position in the trade network is still unaffected [30]. The decoupling of Chinese economy from world financial cycle since 2015 has relatively put China in a slightly better position to handle the adverse impacts of COVID-19. However, it requires extraordinary macroeconomic policies to mitigate the crisis [18].

Against this backdrop, our study contributes towards identification of sectors being potential transmitters/recipients of volatility shocks. This will help regulators to make policies to preclude shocks from being systemic to the stock market. We show that COVID-19 has significantly raised volatility spillovers in India. Also, we find that energy followed by the oil & gas sectors were largest net transmitters of volatility to others while FMCG followed by telecom sectors were largest net recipient of volatility shocks from others. Manufacturing sector didn't show significant volatility co-movement.

## Methodology and data

### Methodology

Method of measuring dynamic spillovers was developed by Diebold and Yilmaz [7]. This technique apportions the forecast error variance (FEV) of a variable to shocks arising from other variables. For any sector index (i), we consider the portion of the FEV due to shocks to sectors (j) other than its own. Then we add the error variances across all other sectors for all i ≠ j.

Diebold-Yilmaz suggested generalized forecast error variance decomposition (GFEVD) technique developed by Koop et al., [17] and Pesaran and Sin [22] to identify the orthogonal structural shocks from the correlated reduced-form shocks. GFEVD is invariant to the ordering of the variables unlike Cholesky factor variance decompositions by considering each variable as first in the ordering. Also, GFEVDs allow for correlated shocks. Variance decompositions can allow us to apportion the H-step ahead FEV of $y_{i}$ due to shocks to other variables i.e., $y_{j}$ for all j≠i, for each i.

Diebold-Yilmaz spillover analysis has three attributes such as the set of variables, H, and the dynamics that allows for time-varying spillovers. The time-varying nature of spillovers is due to evolving tastes, technologies, and institutions. This may also be due to the business cycle, or it may shift abruptly with the onset of financial crisis. We computed daily return series as log changes ($R_{t}=ln\left(\frac{P_{t}}{P_{t-1}}\right)$) in the index closing price. Then volatility series is computed as the five-day rolling window standard deviation of daily return series. The data were obtained from BSE India website which spanned over 1^st^ January 2015 till 9^th^ October 2020.

### Descriptive statistics

The sector indices are namely, auto, bankex, energy, fast moving consumer goods (FMCG), healthcare, IT, manufacturing, oil & gas, realty, and telecom. The descriptive statistics for volatility series are presented in Panel-A of Table 1. Based on Narayan and Popp [21] unit root test, all series were non-stationary except auto and manufacturing sector.^1^ And each series follow normal distribution as evident from the p-values of the Jarque-Bera (JB) statistic. Normality is a pre-requisite for GFEVD.

**Table 1:** Results.

Panel A: summary statistics											
	Mean	Maximum	Minimum	Skewness	Kurtosis	JB	Probability				
Auto	0.011	0.035	-0.016	-0.035	2.924	0.577	0.749				
Bankex	0.011	0.039	-0.019	-0.010	3.046	0.139	0.933				
Energy	0.012	0.037	-0.017	-0.014	2.984	0.058	0.972				
FMCG	0.009	0.027	-0.011	-0.036	2.873	1.162	0.559				
Healthcare	0.010	0.028	-0.010	-0.040	2.906	0.827	0.661				
IT	0.011	0.032	-0.012	-0.032	2.981	0.245	0.885				
Manufacturing	0.009	0.032	-0.014	0.005	2.920	0.356	0.837				
Oil & Gas	0.011	0.036	-0.015	-0.004	2.973	0.043	0.979				
Realty	0.016	0.047	-0.014	0.014	2.889	0.705	0.703				
Telecom	0.014	0.042	-0.015	-0.019	2.917	0.455	0.796				


Panel B: Volatility/Uncertainty spillovers using DY approach											
	Auto	Bankex	Energy	FMCG	Healthcare	IT	Manufacturing	Oil & Gas	Realty	Telecom	From Others
Auto	49.4	13.3	7.0	5.0	4.2	2.4	0.0	8.6	7.3	2.7	50.6
Bankex	12.6	48.1	8.6	3.5	4.8	3.2	0.2	9.2	7.9	1.9	51.9
Energy	6.1	5.8	49.1	2.0	4.8	2.5	0.4	22.4	4.7	2.1	50.9
FMCG	7.5	9.6	4.4	58.1	6.1	1.5	0.0	5.2	5.9	1.7	41.9
Healthcare	4.5	4.2	7.3	5.0	59.9	1.4	0.2	7.8	7.2	2.5	40.1
IT	4.8	4.2	2.6	5.3	3.8	74.5	0.5	2.1	1.8	0.4	25.5
Manufacturing	0.1	0.0	0.0	0.1	0.3	1.2	97.3	0.1	0.0	0.9	2.7
Oil & Gas	7.4	7.6	24.0	3.1	5.8	2.4	0.0	45.1	4.2	0.4	54.9
Realty	6.4	8.3	5.7	3.8	7.2	1.9	0.1	9.1	55.7	1.8	44.3
Telecom	5.0	4.7	4.7	1.0	1.6	1.8	0.5	3.2	2.2	75.2	24.8
Contribution to Others	54.3	57.7	64.3	28.7	38.6	18.4	2.0	67.8	41.2	14.3	387.5
Contribution including Own	103.7	105.8	113.4	86.9	98.6	92.9	99.3	112.8	97.0	89.5	38.7%
Net contribution	3.7	5.8	13.4	-13.2	-1.5	-7.1	-0.7	12.9	-3.1	-10.5	

Panel A of the table reports summary statistics for different sector index volatility series. Whereas Panel B reports the spillover matrix generated through GFEVDs following Diebold and Yilmaz [7] approach. The sample period spans over January 8, 2015 to October 9, 2020. The spillover matrix is estimated at 1 lag, 10 days ahead forecast horizon, and rolling window of 100 days.

## Findings and discussion

### Dynamic volatility spillovers

We have investigated the volatility spillovers among ten sector indices. Our study period has witnessed several major global and regional shocks. These shocks have caused the volatility busts in the Indian stock market. In our analysis, the dynamic (conditional) spillovers account for secular and cyclical movements in the volatility series.

**Fig. 1:**
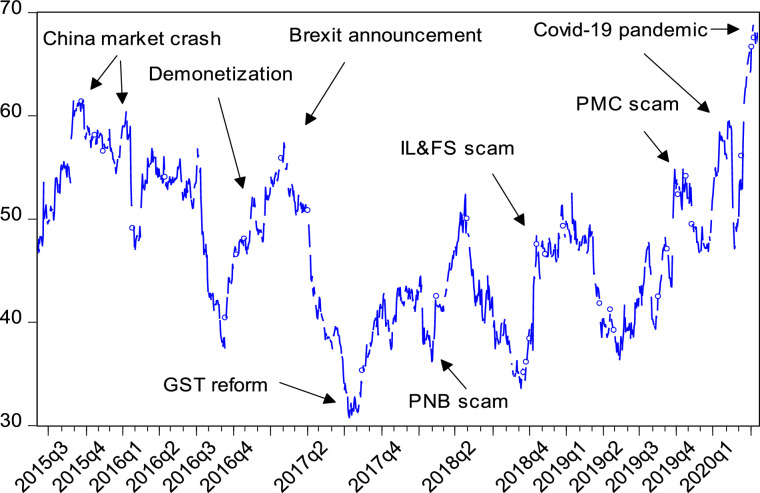
Time-Varying Volatility Spillovers (Sectoral Return Volatility). The figure shows the results of dynamic volatility spillovers between different sector indices. The results are computed by employing the Diebold and Yilmaz [7] model. The sample period spans over January 8, 2015 to October 9, 2020. The plot is estimated at lag=1, H= 10 days, and rolling window= 100 days.

The conditional volatility spillovers across sectors appear as Panel-B in Table 1 and in Fig. 1. The values in Panel-B and total volatility spillover index (TSI) plot in Fig. 1 is estimated over a rolling-window sample of 100 days and a forecast horizon of 10 days. The optimum lag length is 1, based on SIC and HQ criterion.

The TSI value is 38.7% while further decomposition shows important information. We find that oil and gas sector followed by energy remained the largest contributor to the volatility spillovers while the former being the largest recipient of spillovers from others. Energy consumption is considered as an indicator of economic progress and is recognized as a complement to other sectors. Therefore, a shock to energy consumption is likely to have greater impact on the other sectors. The diagonal elements represent its own contributions to the forecast error variance (FEV) due to shock to itself. Manufacturing sector was affected by its own shock rather than being a contributor/recipient. There was negligible spillover impact by/on the manufacturing sector.

Energy sector, followed by oil & gas, bank (bankex), and auto were the net contributors to volatility spillovers. Whereas, FMCG followed by telecom, IT, real estate (realty), and healthcare were net recipient of volatility. Besides, the spread in the ‘contribution to others’ row is larger than ‘contribution from others’ column which means sectors vary largely by their contribution to spillovers.

Fig. 1 depicts the time-varying TSI for volatility series. Spillover plot is showing cyclical patterns with troughs during calmness and peaks during turbulence. Shocks can have an instantaneous or lagged effect on the volatility spillovers. As shown in Fig. 1, volatility busts were induced by the shocks from Chinese stock market crash of 2015-16, demonetization (November, 2016), BREXIT announcement (March, 2017), tax (GST) reform (July, 2017), major defaults and scams like infrastructure leasing and financial services scam (IL&FS, 2018), Punjab and Maharashtra co-operative (PMC, 2019) bank scam, and Punjab National Bank (PNB, 2018) scam and finally the COVID-19 pandemic (2020). The effect of GST and PNB scam on the volatility spillovers includes a time-lag as shown in the plot.

Evidence suggests that volatility spillovers were the highest during COVID-19 pandemic owing to heightened uncertainty and economic activity coming to a standstill. The worldwide impact of the pandemic is deepening and eroding the welfare gains achieved over several decades. What is important is that the final spillover cycle due to the pandemic is far from over as yet the spike has not shown any resistance at the upper level. Thus, total volatility spillover due to the pandemic is the highest (69%) during the study period that witnessed several other shocks. Plots for more disaggregate directional spillovers are not produced here for brevity.

**Fig. 2:**
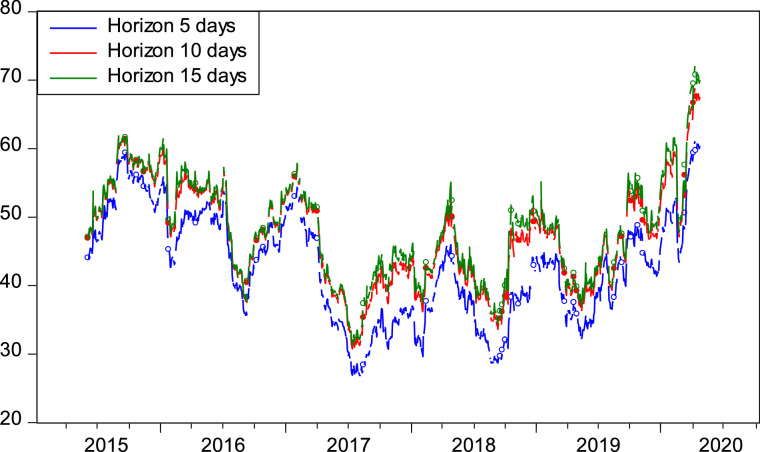
Time-Varying Volatility Spillovers (For Different Horizons). The figure shows the robustness checks on the dynamic sectoral volatility spillovers for different values of forecast horizon. The results are computed by employing the Diebold and Yilmaz [7] model. The sample period spans over January 8, 2015 to October 9, 2020. The lag length and rolling window are fixed at 1 and 100 days, respectively.

**Fig. 3:**
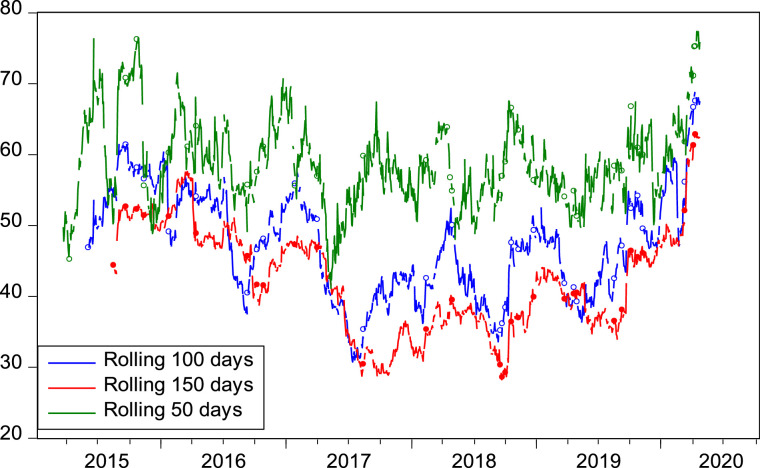
Time-Varying Volatility Spillovers (For Different Rolling Windows). The figure shows the robustness checks on the dynamic sectoral volatility spillovers for different rolling window lengths. The results are computed by employing the Diebold and Yilmaz [7] model. The sample period spans over January 8, 2015 to October 9, 2020. The lag length and forecast horizon are fixed at 1 and 10 days, respectively.

Again, to check the robustness of our main outcome, we computed the volatility spillover index for different forecast horizons such as 5, 10, 15 days (Fig. 2) and different rolling window widths such as 50, 100, 150 days (Fig. 3). We show that TSI plots are insensitive to the choice of both the forecast horizon and window length.

## Conclusion

There is a dearth of studies exploring the volatility/uncertainty spillovers across markets, firms, and sectors in case of India. This study analyzed the risk of contagion between sector indices listed in BSE India by using the variance decompositions in a GVAR framework. All the sectors except manufacturing showed large co-movement at the onset of a crisis. Unlike before, the current situation of intensified uncertainty is unique in the nature of the crisis i.e., health crisis (COVID-19) which has brought forth real and financial crisis. We find that 38.7% of forecast error variance in total return volatility is due to spillovers. Also, we find that energy sector followed by oil & gas were the major net volatility transmitters. The sample period witnessed several shocks, but the uncertainty induced by COVID-19 was the highest. The bursts in volatility spillovers suggest that shocks travel freely and quickly amongst highly integrated sectors.

Our study can help the investors and portfolio managers assess the risk based on the spillover transmission dynamics and make decisions on optimum allocation of assets and portfolio diversification. Investors can benefit by including the stocks from weakly integrated sectors in their portfolio which may reduce their exposure to prolonged uncertainty. Better understanding of spillover dynamics among financial markets, can also help the regulators in assessment of impact of volatility spillovers during adversity.

## Declaration of Competing Interest

The authors declare that they have no conflict of interest

## Appendix Supplementary materials

Image, application 1

## Footnotes

1 The results are not presented here for brevity.
